# Chemokines and matrix metalloproteinases in cerebrospinal fluid of patients with central nervous system complications caused by varicella-zoster virus

**DOI:** 10.1186/s12974-019-1428-1

**Published:** 2019-02-18

**Authors:** Liza Lind, Kristina Eriksson, Anna Grahn

**Affiliations:** 1 Department of Rheumatology and Inflammation Research, Sahlgrenska Academy, University of Gothenburg, Gothenburg, Sweden; 2 Department of Infectious Diseases, Sahlgrenska Academy, University of Gothenburg, Gothenburg, Sweden

**Keywords:** Varicella-zoster virus, Central nervous system infection, Chemokines, Matrix metalloproteinases, Encephalitis

## Abstract

**Background:**

Varicella-zoster virus (VZV) is a common viral agent causing central nervous system (CNS) infections including encephalitis, meningitis, and Ramsay Hunt syndrome. Neurological complications occur frequently despite antiviral treatment. Matrix metalloproteinases (MMPs) and cytokines are involved in the neuroinflammatory response during CNS infection. Their role in VZV CNS infections and how they differ between different CNS entities caused by VZV are poorly investigated.

**Methods:**

We analyzed the levels of 30 chemokines and 9 MMPs in cerebrospinal fluid (CSF) and serum from 66 patients with VZV CNS infections diagnosed by detection of VZV DNA in CSF and concomitant neurological symptoms and compared with a control group (*n* = 24).

**Results:**

Levels of CCL19, CXCL8, CXCL9, and CXCL10 were significantly increased and surpassing the levels in serum when analyzing all patients with VZV CNS infections whereas CXCL11 was only increased in CSF of patients with VZV meningitis. MMP-2-levels were highly elevated in CSF of all 66 VZV patients. The patients with encephalitis had the most significantly increased levels of MMPs in CSF, and MMP-3, MMP-8, and MMP-12 were exclusively increased in this group, whereas MMP-9 in CSF was increased in the patients with VZV meningitis.

**Conclusions:**

We show that both chemokines and MMPs are elevated in the CSF of patients with VZV CNS infections. Encephalitis and meningitis patients differed with respect to other chemokines (CXCL11) and MMPs (MMP-3, MMP-8, MMP-9, and MMP-12), indicating that different location of the virus gives rise to qualitative differences in the ensuing inflammatory response. In addition, the pronounced increase of MMPs in CSF of the patients with encephalitis suggests an association to the severity of this manifestation, compared to VZV meningitis and Ramsay Hunt syndrome. The role of MMPs in association to chemokines should be further investigated to evaluate their significance in the neuropathogenesis of VZV CNS infections and as a potential target for new treatment alternatives.

## Introduction

Varicella-zoster virus (VZV) is recognized as one of the most common viral agents causing central nervous system (CNS) infections and includes a wide spectrum of CNS manifestations such as encephalitis, meningitis, Ramsay Hunt syndrome with facial paralysis, and vasculitis with stroke-like syndromes. Serious neurological complications are reported despite antiviral treatment. Encephalitis caused by VZV is associated with severe neurological sequels with primarily cognitive deficits whereas meningitis and Ramsay Hunt syndrome are considered more benign. The neuropathogenesis of VZV CNS infections is only partly elucidated. After primary infection, VZV establishes latency in sensory ganglia. When VZV reactivates and causes herpes zoster, it is suggested that the virus propagates to CNS primarily from afferent fibers from trigeminal and other ganglia via trans-axonal transport [1]. In the CNS, signs of vessel wall infection have been reported [2, 3]. In only a few cases, the virus has been found in brain parenchyma [4, 5]. How the neurons and the supporting cells of the brain parenchyma are affected by VZV remains poorly defined. The CSF inflammatory response in VZV CNS infections is characterized by pleocytosis, mainly lymphocytes, and increased protein content [6], but in overall terms, the knowledge of the inflammatory response in the CNS caused by VZV is very limited.

The CNS is considered an immune-privileged site. Leukocyte entry into the CNS is restricted by the blood-brain barrier (BBB) and/or the blood-cerebrospinal fluid barrier (BCSFB). However, immune surveillance continuously takes place in the CNS during steady state indicating that leukocyte trafficking over these barriers takes place also in the absence of CNS inflammation [7–9]. During inflammation, lymphocyte trafficking into the CNS accelerates in a multi-step process involving chemokine-induced recruitment of cells across the endothelial brain barriers followed by BBB breakdown, which allows lymphocyte penetration of the parenchymal basement membrane that surrounds the brain and spinal cord [10]. The first step in this process, the secretion profiles in the CNS of humans, is relatively unexplored in viral CNS infections [11]. Best described are CXCL9, CXCL10, and CXCL11, which bind CXCR3, a chemokine receptor primarily expressed on T cells and natural killer (NK) cells [12, 13]. High levels of CXCL9 and CXCL10 are detected in CSF of herpes simplex encephalitis and meningitis patients, whereas CXCL11 is exclusively found in herpes simplex meningitis patients [14]. In entero- and paramyxoviral meningitis, the CSF levels of CXCL10, but not CXCL9, are high [15]. Very little is known regarding chemokine secretion patterns in the CSF of VZV patients.

The second step in the process, the BBB breakdown, may be due to direct trauma or mediated by inflammatory modulators [7–9] such as matrix metalloproteinases (MMPs) that cause basement membrane degradation, cleavage of tight junction proteins, and detachment of astrocytes from the parenchymal basement membrane [16–18]. MMPs are part of the inflammatory response to CNS infections, and many cell types in the CNS are able to produce MMPs [18]. MMPs are zinc-dependent enzymes with proteolytic activity that acts on the extracellular matrix. MMP-2, MMP-3, and MMP-9 have all been associated with the neuroinflammatory response [19]. MMP-3 and MMP-9 are inducible MMPs involved in inflammatory responses in the CNS, whereas MMP-2 is constitutively expressed by astrocytes and activated upon host response to injury. MMP-2, MMP-3, and MMP-9 all cleave tight junction proteins thus contributing to BBB breakdown [18, 20]. Recent studies have shown increased concentrations of MMP-1, MMP-2, MMP-3, MMP-9, and MMP-10 in the CSF of patients with VZV CNS vasculopathy [21], and an association between increased MMPs and vasculitis in these patients has been suggested. The presence and patterns of different MMPs and chemokines in patients with other VZV CNS infections than VZV vasculopathies have not yet been elucidated.

Our aim was to investigate the concentrations of 30 chemokines and 9 MMPs in the CSF and serum of patients with VZV CNS infections diagnosed by VZV DNA by PCR in the CSF and to correlate the amount of these chemokines and MMPs to clinical entities and parameters. We found that CXCL8, CXCL9, CXCL10, CXCL11, and CCL19 were significantly increased in CSF to levels above those found in serum. In addition, CSF levels of MMP-2, MMP-3, MMP-8, MMP-9, and MMP-12 reached high to very high levels in patients with VZV CNS infections compared to controls. Most pronounced were the increased levels of MMPs in patients with encephalitis, indicating an association to the severity of this manifestation, compared to VZV meningitis and Ramsay Hunt syndrome.

## Materials and methods

### Study population and procedures

Patients from the region of Västra Götaland in Sweden, with VZV DNA detected in the CSF by real-time PCR between 2004 and 2015, were retrospectively identified at the Laboratory of Virology at Sahlgrenska University Hospital, Gothenburg, Sweden. The inclusion criteria were contemporary acute CNS symptoms in patients categorized as encephalitis, meningitis, or Ramsay Hunt syndrome, based on previously presented criteria [22], and available samples of CSF. Clinical symptoms of encephalitis were fever and altered mental status or focal neurological deficits, of meningitis; fever, headaches, nausea, and light and sound sensibility and of Ramsay Hunt syndrome; facial palsy; and sometimes hearing deficiencies. The medical records were obtained for registration of clinical data including laboratory results and treatment. All patients with CNS symptoms were assessed as having reactivated VZV infections based on patients’ awareness of previous varicella infection, age, and clinical symptoms. Antiviral treatment was given according to national recommendations in Sweden, i.e., in encephalitis and severe cases of Ramsay Hunt syndrome, 10–15 mg/kg t.i.d of acyclovir is given for 7–14 days. Patients with meningitis are recommended oral treatment with 1 g t.i.d of valacyclovir for 7 days (Health Care Program for Viral CNS Infections, Sweden, 2015). Patients were lumbar punctured for CSF and sampled for serum at the time for acute disease as a part of routine clinical procedures. Blood was drawn into polypropylene tubes and then centrifuged 4000 rpm for 10 min to receive serum collected in polypropylene tubes. Some CSF samples were centrifuged before storage, and all CSF samples were centrifuged 1500 rpm for 5 min to remove cells after storage and before further analysis. Both CSF and serum samples were stored at − 70 °C. From the patients, paired CSF and serum samples (± 2 days) were selected for further analysis.

CSF and serum samples from 24 non-infectious persons that had sought care for headaches were included. All control subjects had normal neurological status at examination, normal CSF cell count, and normal CSF albumin concentration. None of them were diagnosed with any neurological or other diseases that could be presumed to affect the CNS, within 1 year after sampling.

### Analysis of viral load, cell counts, and albumin levels in CSF and serum

The CSF samples were analyzed for VZV DNA by a quantitative in-house TaqMan PCR method as described previously [6] at the time of acute disease. Automated CSF cell counting was performed on a Siemens ADVIA 2120i instrument within 1 h of sampling using the ADVIA 2120i CSF Assay according to the manufacturer’s instructions (Siemens AG, Erlangen, Germany). Albumin levels in CSF and serum were measured by immunonephelometry on a Beckman Immage Immunochemistry system (Beckman Instruments, Beckman Coulter, Brea, CA, USA), and the albumin ratio was calculated (CSF albumin (mg/l)/serum albumin (g/l)) and used as an indicator of blood-brain barrier damage.

### Analysis of chemokines and MMPs

Chemokines (CCL1, CCL2, CCL3, CCL7, CCL8, CCL11, CCL13, CCL15, CCL17, CCL19, CCL20, CCL21, CCL22, CCL23, CCL24, CCL25, CCL26, CCL27, CX3CL1, CXCL1, CXCL2, CXCL5, CXCL6, CXCL8, CXCL9, CXCL10, CXCL11, CXCL12, CXCL13, CXCL16) and MMPs (MMP-1, MMP-2, MMP-3, MMP-7, MMP-8, MMP-9, MMP-10, MMP-12, and MMP-13) were detected and quantified using a human magnetic Luminex assay (Bio-Plex Pro™ Assay; Bio-Rad Laboratories) according to the manufacturer’s instructions and analyzed using Bio-Plex 200 system (Bio-Rad) with five-parameter logistic standard curves which were used for interpolation of chemokine and MMP levels.

### Statistical analysis

Mann-Whitney *U* test was used for comparisons between two groups, and Kruskal Wallis non-parametric test with Dunn’s post-test was used for multi-group comparisons. Non-parametric Spearman’s correlation coefficient test was used for correlations. Statistical analyses were performed using GraphPad Prism version 7 (GraphPad Software). One patient with VZV meningitis and vasculitis as a result of systemic lupus erythematosus showed extremely deviating results, that is why she was regarded as an outlier and was not included in further statistical analysis to avoid misleading data.

## Results

### Patients and sampling

Seventy-two patients with VZV DNA detected in the CSF were included. In 67 patients, sufficient amount of frozen CSF was available for further analysis. These 67 patients were categorized into the following diagnostic entities: encephalitis (*n* = 29), meningitis (*n* = 21), and Ramsay Hunt syndrome (*n* = 17). In 34 of these 67 patients, paired CSF and serum samples were analyzed (encephalitis (*n* = 11), meningitis (*n* = 14), Ramsay Hunt syndrome (*n* = 9)). Thirty of 34 serum samples were drawn the same day as the CSF and the other four were drawn within 3 days. Lumbar punctures were performed median 4 days (range 0–90) after onset of neurological symptoms. Four patients with encephalitis had changes at computer tomography (CT) and/or at magnetic resonance imaging (MRI) of the brain compatible with acute ischemic stroke, which were regarded as VZV manifestations. One other patient with encephalitis had concomitant symptoms of polyneuropathy. Clinical data are presented in Table 1. Six patients were immunocompromised (chronic lymphatic leukemia (*n* = 1), rheumatoid arthritis (*n* = 1), prostate malignancy (*n* = 1), systemic lupus erythematosus (*n* = 1), vasculitis (*n* = 1), and ulcerous colitis (*n* = 1)). All received immunosuppressive treatment. Antiviral treatment was administered median 0 day (range 5–6) after lumbar puncture.

**Table 1:** Clinical data of patients with VZV CNS infections and controls (median and range)

	Encephalitis	Meningitis	Ramsay Hunt	Controls
Patients no.	29	21	17	24
Age	75 (29–88)	30 (15–84)	60 (13–82)	38 (17–76)
F/M	10/18	9/12	6/11	10/14
CSF VZV DNA copies/ml	17,000 (70–25 millions)	15,100 (50–224,000)	950 (50–759,000)	NA
Intravenous treatment^1^ (days) (no.)	11 (0–18) (no. 27)	2 (1–10) (no. 14)	8 (2–14) (no. 10)	NA
Oral treatment^2^ only (days) (no.)	(no. 2)	7 (7–7) (no. 6)	7 (7–14) (no. 8)	NA

*VZV* varicella-zoster virus, *CNS* central nervous system, *CSF* cerebrospinal fluid, *NA* not analyzed, *t.i.d* three times/day

^1^Dosage of i.v acyclovir was 10–15 mg/kg t.i.d, except for patients with renal failure. Additionally, oral treatment to the i.v treatment was given to 23 patients with VZV CNS infections with valacyclovir 1 g t.i.d or acyclovir 800 mg five times a day

^2^Oral treatment was given as valacyclovir 1 g t.i.d

### Viral load and cells in different VZV CNS entities

**Fig. 1:**
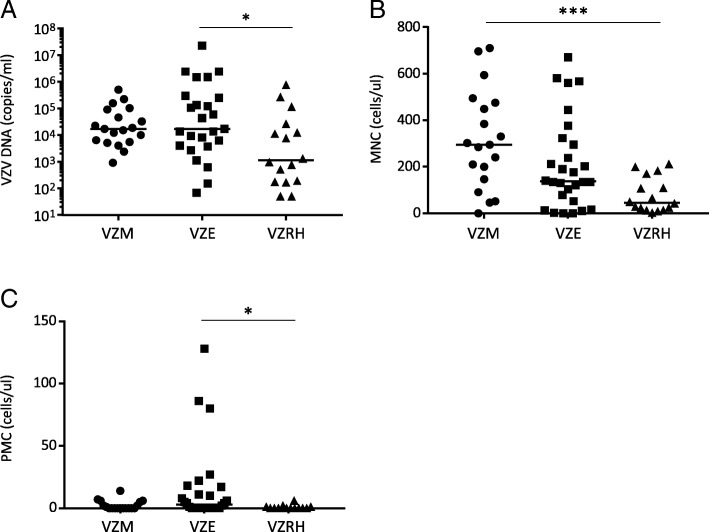
Levels of virus and cells in CSF from patients with VZV CNS infections. VZV DNA copies (**a**), MNC numbers (**b**), and PMNC numbers (**c**) in the CSF were compared in VZV patients with different CNS disease manifestations: encephalitis (*n* = 29), meningitis (*n* = 21), and Ramsay Hunt syndrome (*n* = 17). Data are presented as individual values with medians indicated by horizontal bars. Comparisons were performed using Kruskal-Wallis’ non-parametric test with Dunn’s post-test. ***p < 0.001, *p < 0.05

The viral load in CSF that were measured during routine clinical analysis by PCR varied considerably between different individuals, but overall, the levels were higher in patients with encephalitis compared to patients with Ramsay Hunt syndrome (Table 1 and Fig. 1a). Meningitis was associated with higher levels of mononuclear cells (MNC) in CSF, as previously described, whereas encephalitis was associated with higher levels of polymorphonuclear leukocytes (PMN), compared to patients with Ramsay Hunt syndrome (Fig. 1b, c).

### Increased levels of CCL19, CXCL8, CXCL9, CXCL10, and CXCL11 in VZV CNS infections

**Fig. 2:**
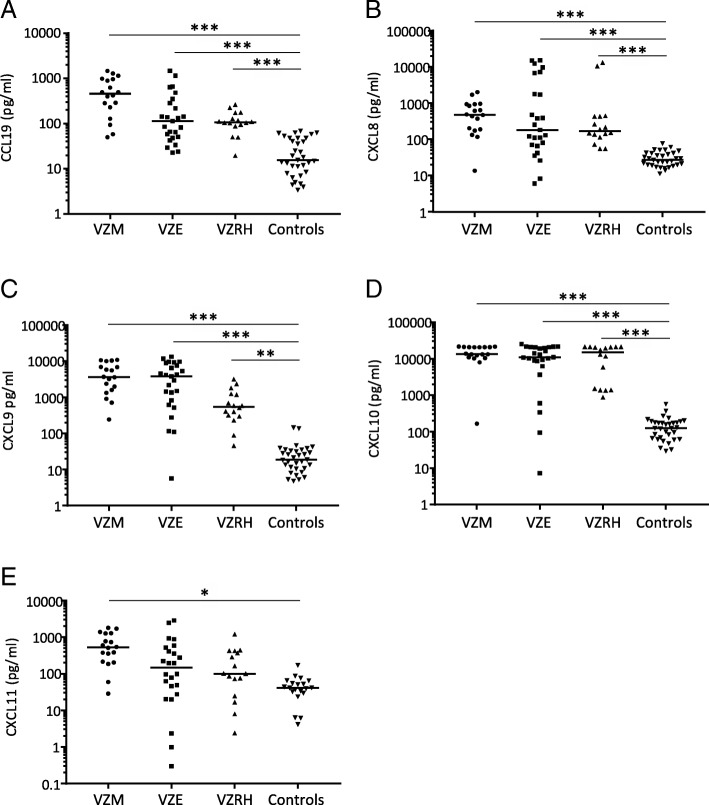
Chemokine levels in CSF from patients with VZV CNS infections. CSF levels of CCL 19 (**a**), CXCL8 (**b**), CXCL9 (**c**), CXCL10 (**d**), and CXCL11 (**e**) in the CSF were compared in VZV patients with different CNS disease manifestations: encephalitis (*n* = 25), meningitis (*n* = 19), and Ramsay Hunt syndrome (*n* = 16) and in uninfected controls (*n* = 19). Data are presented as individual values with medians indicated by horizontal bars. Comparisons were performed using Kruskal-Wallis’ non-parametric test with Dunn’s post-test. ***p < 0.001, **p < 0.01, *p < 0.05

We analyzed the levels of 30 different chemokines in CSF and, when available, in serum from 60 of the 67 VZV patients and 21 of the 24 control patients. Twenty-six of the 30 chemokines were significantly elevated in CSF from VZV patients; only CCL2, CCL21, CXCL12, and CXCL16 did not increase upon VZV CNS infection (not shown). However, when we adjusted these data to the levels found in serum from the VZV patients, we identified five chemokines that were not only increased in the CSF of VZV patients but also reached levels in the CSF surpassing those found in serum, thus creating a chemotactic gradient towards the CNS. CCL19, CXCL8, CXCL9, and CXCL10 were significantly increased compared to CSF from control patients and compared to levels in serum in patients with encephalitis, meningitis, and Ramsay Hunt syndrome (Fig. 2a–d) whereas CXCL11 was only increased in CSF in VZV meningitis patients (Fig. 2e). Overall, CXCL9 and in particular CXCL10 reached very high concentrations in the CSF. We could not detect any significant differences in chemokine concentrations when comparing patients with encephalitis, meningitis, and Ramsay Hunt syndrome.

The CSF levels of CXCL8 correlated both to the viral load in the CSF (*p* = 0.004) and to the number of infiltrating PMN (*p* = 0.002) which is consistent with the fact that virus levels, PMN, and CXCL8 were all enhanced in VZV encephalitis patients. There was a strong correlation between the CSF levels of CCL19 and the number of MNC in the CNS (*p* = 0.0003). Weak associations were found between CXCL9 and numbers of MNC (*p* = 0.020) and CXCL11 and PMN (*p* = 0.046).

### Increased levels of MMP-2, MMP-3, MMP-8, MMP-9, and MMP-12 in VZV CNS infections

**Fig. 3:**
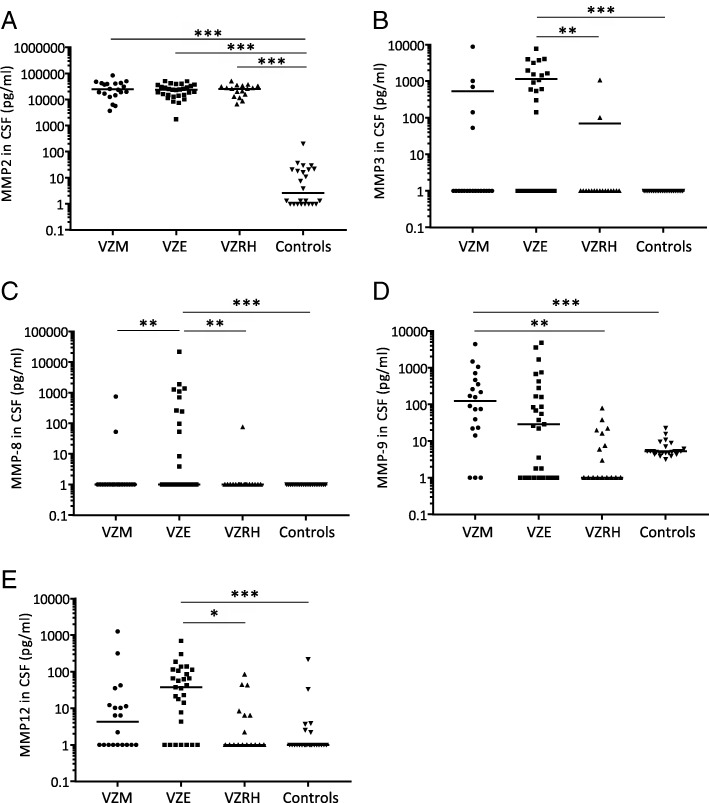
MMP levels in CSF from patients with VZV CNS infections. CSF levels of MMP2 (**a**), MMP3 (**b**), MMP8 (**c**), MMP9 (**d**), and MMP12 (**e**) were compared in VZV patients with different CNS disease manifestations: encephalitis (*n* = 29), meningitis (*n* = 20), and Ramsay Hunt syndrome (*n* = 17) and in uninfected controls (*n* = 24). Data are presented as individual values with medians indicated by horizontal bars. Comparisons were performed using Kruskal-Wallis’ non-parametric test with Dunn’s post-test. ***p < 0.001, **p < 0.01, *p < 0.05

**Fig. 4:**
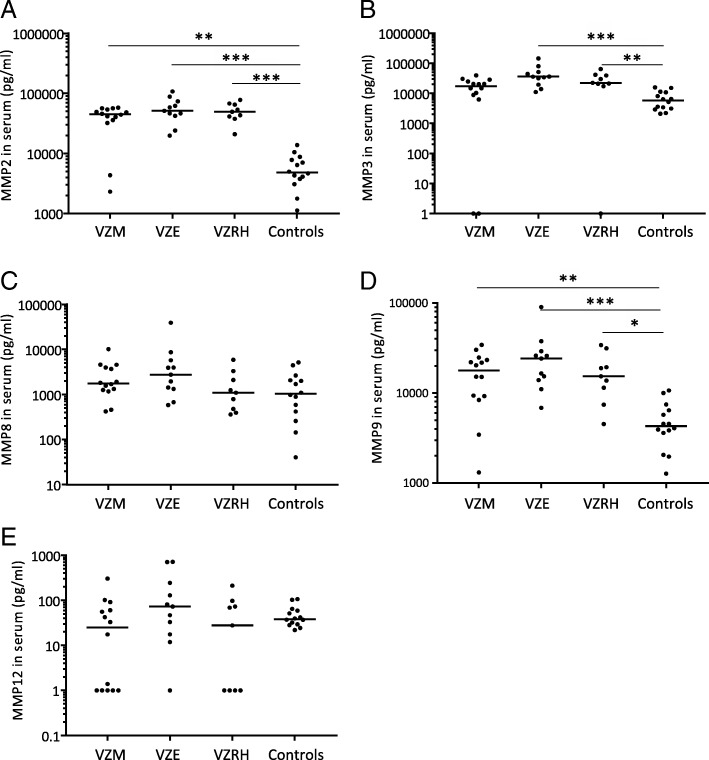
MMP levels in serum from patients with VZV CNS infections. Serum levels of MMP2 (**a**), MMP3 (**b**), MMP8 (**c**), MMP9 (**d**), and MMP12 (**e**) were compared in VZV patients with different CNS disease manifestations: encephalitis (*n* = 11), meningitis (*n* = 14), and Ramsay Hunt syndrome (*n* = 9) and in uninfected controls (*n* = 14). Data are presented as individual values with medians indicated by horizontal bars. Comparisons were performed using Kruskal-Wallis’ non-parametric test with Dunn’s post-test. ***p < 0.001, **p < 0.01, *p < 0.05

We analyzed the levels of nine different MMPs (MMP-1, MMP-2, MMP-3, MMP-7, MMP-8, MMP-9, MMP-10, MMP-12, MMP-13) in CSF and, when available in serum, from 67 VZV patients and 24 control patients. MMP-2-levels increased 10,000-fold in CSF from patients with encephalitis, meningitis, and Ramsay Hunt syndrome and was the only MMP to be increased in all disease entities and also in CSF from all 67 patients (Fig. 3a). VZV encephalitis patients also had significantly elevated levels of MMP-3, MMP-8, and MMP-12 in CSF, whereas VZV meningitis patients instead had significantly elevated levels of MMP-9 (Fig. 3b–e). These results were partly reflected in serum (Fig. 4a–e). Levels of MMP-2 were increased in serum from all but two VZV patients (Fig. 4a). Serum levels of MMP-9 were significantly increased in encephalitis, meningitis, and Ramsay Hunt syndrome patients (Fig. 4d) whereas serum MMP-3 levels were higher in meningitis and Ramsay Hunt syndrome compared to controls (Fig. 4b). No correlations were seen between serum and CSF levels of MMP-2, MMP-3, MMP-8, MMP-9, and MMP-12. To investigate the influence of BBB damage on MMP levels, we correlated the albumin ratios (CSF albumin (mg/l)/serum albumin (g/l)) (data retrieved from routine clinical analysis) to the MMP ratios (CSF MMP (pg/ml)/serum MMP (pg/ml)). A modest correlation was shown between albumin ratios and MMP-8 and MMP-9 (*p* = 0.034 and *p* = 0.031 respectively).

When analyzing cell numbers in the CSF, there was a very strong correlation between the CSF levels of MMP-9 and the number of MNC in the CNS (*p* < 0.0001). No other correlations were shown between the number of MNC and the CSF levels of the other MMPs. The number of PMN in CSF was highly associated with the increased levels of MMP-8 in CSF (*p* = 0.0001) and to a minor extent with the levels of MMP-3 (*p* = 0.002) and MMP-9 (*p* = 0.032). A moderate correlation between viral load in CSF and the levels of MMP-3 in CSF in the VZV patients was shown (*p* = 0.007) which was slightly more pronounced in the patients with meningitis (*p* = 0.002). No other correlations were found between viral load in CSF and concentrations of MMPs.

### Influence of other clinical parameters on levels of MMPs and chemokines

To evaluate other variables that could have influenced the results, we also analyzed clinical parameters besides the ones already mentioned (viral load and cells).

When comparing patients with blisters (*n* = 44) and without blisters (*n* = 21) in the VZV patients, an inverse relation was revealed with moderate higher concentrations of MMP-9 in CSF of patients lacking blisters compared to patients with blisters (*p* = 0.008). In serum, there were no significant differences in MMP levels or chemokine levels (CCL19, CXCL8, CXCL9, CXCL10, CXCL11) depending on if the patients had blisters or not.

As there were differences in age between the subgroups (see Table 1) with significant older patients in the group of encephalitis compared to meningitis (*p* < 0.0001) and Ramsay Hunt syndrome (*p* = 0.022) and also a modest difference between VZV patients as a whole group compared to controls (*p* = 0.025), we also correlated age to the levels of MMPs and chemokines. In CSF, only MMP-12 of the five increased MMPs in CSF (MMP-2, MMP-3, MMP-8, MMP-9, and MMP-12) correlated with age (*p* < 0.001). In serum, levels of MMP-3 and MMP-12 correlated with age (*p* = 0.046 and *p* < 0.001 respectively). However, the associations to age disappeared when the patients were subgrouped. No correlations were shown between age and chemokine levels (CCL19, CXCL8, CXCL9, CXCL10, CXCL11) in CSF.

Modest increased concentrations of MMP-8 in CSF were expressed in the immunocompetent patients (*n* = 60) compared to the immunocompromised patients (*n* = 5) (*p* = 0.009); otherwise, there were no significant differences in MMP levels or chemokine levels (CCL19, CXCL8, CXCL9, CXCL10, CXCL11) in CSF depending on immune status.

No significant association was shown between chemokine levels (CCL19, CXCL8, CXCL9, CXCL10, CXCL11) in CSF or MMPs in CSF or serum and numbers of days between onset of symptoms and CSF sampling (not shown). Taken together, the analyzed possible confounding variables can only have influenced the results in very minor extent.

## Discussion

This is to our knowledge the first study that investigates chemokines and MMPs in patients with various CNS manifestations caused by VZV infection. We demonstrate that CNS complications caused by VZV virus are associated with highly elevated CSF levels of the chemokines CCL19, CXCL8, CXCL9, CXCL10, and the matrix metalloproteinase MMP-2 in patients with different CNS manifestations such as encephalitis, meningitis, and Ramsay Hunt syndrome. In addition, patients with encephalitis had pronounced increased levels of MMP-3, MMP-8, and MMP-12 in their CSF compared to controls, suggesting an association to the severity of this manifestation.

Prior to this study, little was known regarding the chemokine responses during VZV infection in humans neither in the systemic compartment nor in the CNS. We found that VZV CNS complications led to significantly increased CSF levels of 26 out of 30 examined chemokines. Elevated levels of CXCL13 have previously been identified in CSF fluid from two patients with VZV meningitis [23], which we confirm. However, CXCL13, like 20 of the other chemokines that were significantly increased in CSF from patients with VZV CNS disease, never reached concentrations in CSF that surpassed those found in serum and could thus not create a positive CSF to blood chemokine gradient. The biological significance of these chemokines for leukocyte recruitment into the CNS therefore remains uncertain. Only CCL19, CXCL8, CXCL9, CXCL10, and CXCL11 reached concentrations in the CSF that were higher than those found in serum samples from the VZV patients.

VZV can induce CXCL8 production in a variety of different cell types in vitro [24, 25]. We found high levels of CXCL8 in the CSF following VZV CNS infection and that levels of CXCL8 correlated to the CSF viral load, indicating a dose-dependent CXCL8 response to the virus. CXCL8 is the main chemoattractant for neutrophils [26], the most abundant subset of PMN. High levels of CXCL8 have been reported in several other viral CNS infections including herpes simplex encephalitis, herpes simplex meningitis, and Japanese encephalitis patients [14, 27, 28]. Increased levels of CXCL8 in the CSF have been correlated with PMN influx in many forms of bacterial meningitis [29]. Our data show that this is true also in VZV CNS infections as CXCL8 levels in CSF correlated to the numbers of PMNs found in the CSF.

CCL19 binds the CCR7 receptor expressed on T cells and B cells and is one of several chemokines that are constitutively expressed in the CNS [14, 30]. During steady state, CCL19 is involved in the homeostatic surveillance of the CNS by CCR7-positive memory CD4+ T cells [31]. VZV CNS infection led to an accelerated CSF production of CCL19 which correlated with enhanced numbers of MNC in the CSF. Studies in mice show that neurotropic virus infection activates the production of CCL19 which is a crucial requirement for recruitment of protective antiviral CD8+ T cells to the CNS [32, 33]. Our data imply that CCL19 might also in humans represent an important first step in T cell recruitment to the CNS during VZV CNS infection.

We found high levels of CXCL9 and CXCL10 in CSF from patients with VZV CNS complications. VZV infection leads to CXCL10 production in a variety of cell types [24, 25] including human dorsal root ganglia [34]. The significance of CXCL10 for T cell recruitment to the CNS during VZV infections comes from studies in macaques infected with the closely related simian varicella virus, where brain levels of CXCL10 correlate with numbers of infiltrating CD8+ T cells [35]. CXCL9 has not been studied previously in the context of human VZV infection. CXCL9 and CXCL10 are hallmarks of many viral CNS infections including encephalitis caused by HSV-1 and meningitis caused by HSV-2 [14, 27, 36]. Both these chemokines bind CXCR3 on effector T cells and NK cells [12, 13], leading to their recruitment into the infected CNS. The third CXCR3-binding chemokine is CXCL11 [12, 13] which is found in the CNS during, e.g., enteroviral meningitis [37]. We have previously shown that CXCL11 production distinguishes herpes simplex meningitis from herpes simplex encephalitis [14]. The same holds true for VZV as VZV meningitis patients but not patients with VZV encephalitis or Ramsay Hunt syndrome had significantly elevated CXCL11 CSF levels [14]. However, the CXCL11 levels found in VZV meningitis were relatively modest compared to CXCL9 and CXCL10 levels and also compared to CXCL11 levels in herpes simplex meningitis [14]. Yet, the combined data imply that CXCL11 might be a hallmark of viral meningitis.

The increase of the constitutively produced MMP-2 may disrupt basal lamina and tight junctions between endothelial cells, leading to BBB disruption. We found a 10,000-fold increase of MMP-2 in CSF from patients with encephalitis, meningitis, and Ramsay Hunt syndrome which supports a previous study of VZV patients with CNS vasculopathies that also showed high levels of MMP-2 in CSF [21]. Animal models of viral encephalitis have shown associations between increased concentrations of MMP-2 and infected brain cells such as astrocytes, microglia, and endothelial cells [38]. In animal models of herpes simplex encephalitis, MMP-2 levels in CSF were high early in infection, as well as levels of MMP-9 [39]. In addition, an elevation in MMP-2 in the early stages of cerebral ischemic injury has been observed in rodents and nonhuman primates [40]. Although released by several cells of the neurovascular unit, MMP-2 seems to have a predilection for astrocytes which have MMP-2 in their end feet that surround the endovascular cells and neurons [19]. Previous studies on VZV CNS patients have shown astrogliosis interpreted as oxidative stress on astrocytes [22, 41]. Thus, it is possible that besides BBB disruption, the increased MMP-2 levels in CSF of VZV patients can also be associated with the increased activity of astrocytes exposed to oxidative stress.

Only the patients with VZV meningitis demonstrated increased levels of MMP-9 in CSF. In humans, high levels of MMP-9 have been found in the CSF in both viral and bacterial meningitis [29, 42]. In the early stage of the disease, neutrophils and macrophages are the most important source of MMP-9 [29, 43], which is supported by our results that showed a strong association between MMP-9 levels in CSF and MNC and to a lesser extent to PMN. The role of BBB impairment and the association to increased MMPs in CSF in bacterial and viral meningitis has been discussed. It has been suggested that the elevated MMP-9 levels are primarily related to the number of immigrated leucocytes and in less degree to BBB impairment [43]. However, although no correlation was shown between serum and CSF levels of MMP-9 (or any other MMP), the correlation of BBB impairment to the MMP CSF/serum ratio and the fact that the MMP-9 levels in serum were considerably higher than those in CSF indicate that a portion of the MMP-9 in CSF was serum derived.

Also, MMP-2 and MMP-3 were increased in serum of the VZV patients. As for MMP-9, we cannot exclude that a portion of these MMPs in CSF may be derived from serum.

The patients with VZV encephalitis had elevated levels of MMP-3, MMP-8, and MMP-12 in CSF compared to controls, which may reflect a more intense neuroinflammatory response in these patients compared to the other groups. MMP-3 is an intercellular signaling molecule that modulates neuroinflammatory responses [44]. Both stressed and apoptotic neurons release MMP-3, which triggers microglial activation and production of inflammatory cytokines [19]. Subsequently, the elevated levels of MMP-3 in the VZV encephalitis patients may be associated with the neuronal injury shown in VZV CNS infections, reported to be most pronounced in patients with VZV encephalitis [22]. Moreover, as for CXCL8, MMP-3 levels in CSF correlated to the viral load, suggesting a dose-dependent release of MMP-3.

Both MMP-8 and MMP-12 have been reported as critical factors for brain damage in cerebral ischemia in animal studies [45, 46]. MMP-12 is upregulated several-fold higher than any other MMP tested after focal cerebral ischemia. VZV encephalitis is proposed to be primarily a vasculopathy [1], and an increased risk of stroke within 1 year after herpes zoster has been shown in recent studies [47–49]. A potential association between these upregulated MMPs and vasculopathies caused by VZV cannot be ruled out.

## Conclusions

The present study demonstrates that patients with VZV CNS infections have a high CSF production of CXCL8 which attracts neutrophils, CCL19, CXCL9, and CXCL10, which attracts virus-specific T cells, and MMP-2 that facilitates the breakdown of the BBB and thus cell infiltration into the parenchyma. In addition, patients with encephalitis showed pronounced increased levels of MMP-3, MMP-8, and MMP 12 in their CSF compared to controls, indicating an association to the severity of this manifestation. The role of MMPs in association to chemokines should be further investigated to evaluate their role in the neuropathogenesis of VZV CNS infections and as a potential target for new treatment alternatives.

## Abbreviations

BBB: Blood-brain barrier
BCSFB: Blood-cerebrospinal fluid barrier
CNS: Central nervous system
CSF: Cerebrospinal fluid
CT: Computer tomography
HSV: Herpes simplex virus
HSV-1: Herpes simplex virus type 1
HSV-2: Herpes simplex virus type 2
MMP: Matrix metalloproteinase
MNC: Mononuclear cells
MRI: Magnetic resonance imaging
NK: Natural killer
PML: Polymorphonuclear leukocytes
VZV: Varicella-zoster virus
